# The association between body mass index and neoadjuvant chemotherapy response in patients with breast cancer

**DOI:** 10.1186/s13058-025-02083-w

**Published:** 2025-07-11

**Authors:** Jonas Busk Holm, Stine Blaabjerg Skovbjerg, Hanne Melgaard Nielsen, Peer Christiansen, Jens Meldgaard Bruun, Jan Alsner, Deirdre Cronin-Fenton, Signe Borgquist

**Affiliations:** 1 Department of Clinical Medicine, Aarhus University, Aarhus, Denmark; 2 Department of Oncology, Aarhus University Hospital, Aarhus, Denmark; 3 Department of Plastic and Breast Surgery, Aarhus University Hospital, Aarhus, Denmark; 4 Steno Diabetes Center Aarhus, Aarhus University Hospital, Aarhus, Denmark; 5 Department of Experimental Clinical Oncology, Aarhus University Hospital, Aarhus, Denmark; 6 Department of Clinical Epidemiology, Aarhus University Hospital, Aarhus, Denmark

**Keywords:** Neoadjuvant chemotherapy, Obesity, Overweight, Pathological complete response, Body mass index

## Abstract

**Background:**

Obesity, defined as Body Mass Index (BMI) ≥ 30 kg/m^2^, is associated with inferior breast cancer prognosis, but its effect on neoadjuvant chemotherapy response is uncertain. We hypothesized that obesity decreases the odds of pathological complete response (pCR) after neoadjuvant chemotherapy.

**Methods:**

We assembled a cohort of women with breast cancer who underwent neoadjuvant chemotherapy and subsequent surgery between January 1, 2016, and December 31, 2020, in Denmark. Patients received six or eight series of EC-TAX (epirubicin, cyclophosphamide, and paclitaxel) based on disease stage. Trastuzumab and pertuzumab were also used for patients with HER2+ disease. BMI was assessed as a categorical variable (normal weight (BMI = 18.5-<25 kg/m^2^), overweight (BMI = 25-<30 kg/m^2^), and obesity (BMI ≥ 30 kg/m^2^)) and as a continuous variable. We used multivariable logistic regression models to compute odds ratios (ORs) for pCR after neoadjuvant chemotherapy according to BMI groups, using normal weight as reference, and stratified by menopausal, estrogen receptor (ER), and HER2 status. We adjusted for age and menopausal status based on a directed acyclic graph.

**Results:**

Among 1819 patients, 417 had pCR. Patients with overweight (*N* = 585) or obesity (*N* = 450) had 22% and 27% lower odds, respectively, of pCR (OR_adj_=0.78 [95%CI = 0.60-1.00] and OR_adj_=0.73 [95%CI = 0.55–0.97]) compared with patients with normal weight (*N* = 784). In ER/HER2-stratified analyses, we observed lower pCR odds among women with obesity and HER2+ tumors (OR_adj_=0.72 [95%CI = 0.47–1.12]) compared with their normal weight counterparts, but no notable association appeared for ER+/HER2- (OR_adj_=0.97 [95%CI = 0.49–1.96]) and ER-/HER2- tumors (OR_adj_=0.88 [95%CI = 0.49–1.57]). In analyses stratified by menopausal status, obesity was associated with lower pCR odds among postmenopausal women (OR_adj_=0.62 [95%CI = 0.41–0.94]), and, to a lesser extent, premenopausal women (OR_adj_=0.86 [95%CI = 0.58–1.27]).

**Conclusions:**

Our findings suggest that breast cancer patients with overweight or obesity have lower odds of pCR compared with patients with normal weight. As the results varied by ER and HER2 status, the observed association may depend on subtype. In summary, our results are consistent with earlier studies that propose BMI as a potential prognostic marker of pCR.

**Supplementary Information:**

The online version contains supplementary material available at 10.1186/s13058-025-02083-w.

## Background

Breast cancer is the most common cancer among women worldwide (excluding non-melanoma skin cancer) and the leading cause of cancer deaths for women [1]. In 2022, 2.3 million women were diagnosed with breast cancer, and more than 650,000 women died from the disease [2]. Concurrently, as of 2022, more than 890 million adults worldwide were living with obesity (body mass index (BMI) ≥ 30 kg/m^2^) according to the World Health Organization [3]. Obesity is associated with an increased risk of at least 15 types of cancer, including breast cancer in postmenopausal women, as well as increased risk of recurrence and mortality among patients with breast cancer [4–6]. Therefore, a challenge lies in outlining the optimal treatments for patients with obesity and breast cancer to improve their prognosis.

Breast cancer is treated multimodally. Neoadjuvant chemotherapy (NACT) was introduced in the 1970s and was used to downstage inoperable breast cancer to operable [7, 8]. In current practice, NACT is also used for operable breast cancer, as it can increase the possibility of breast-conserving surgery and decrease the number of patients needing axillary dissection [9–11]. Importantly, in operable breast cancer, the decision to administer chemotherapy either before or after breast surgery does not affect long-term clinical outcomes [9, 12].

The primary outcome measure after NACT is pathological response, and the preferred outcome is pathological complete response (pCR), which is defined as total regression of invasive cancer in the breast and lymph nodes [13]. The achievement of pCR is associated with improved long-term outcomes, particularly among patients with triple-negative breast cancer or Human Epidermal Growth Factor Receptor 2 positive/hormone-receptor-negative (HER2+/HR-) tumors [13]. Tumor characteristics, such as receptor status, histological grade, histological classification, and Ki67 are associated with the likelihood of achieving pCR [13–15], but the importance of BMI remains unclear.

Studies investigating the association between BMI and NACT have had ambiguous results [16–28]. A meta-analysis by Wang et al. [16] from 2021 concluded that patients with overweight (BMI = 25-<30 kg/m^2^) or obesity were less likely to achieve pCR compared with patients with BMI < 25 kg/m^2^. Most of the studies included in the meta-analysis found that such patients had lower odds of pCR, but some studies found no association. The most recent studies have found differing results as well [21–28], with one study even reporting higher odds of achieving pCR for patients with obesity [28]. In addition, conflicting results have been reported in studies on the association between BMI and NACT response stratified by receptor status [19, 25–27, 29–31] and menopausal status [17, 22, 25, 27, 31].

As such, the association between BMI and the achievement of pCR remains unclear, and it could be beneficial to incorporate such information in clinical decision-making. Furthermore, it is unclear how this association differs according to receptor status and menopausal status. Therefore, we investigated the overall association between BMI and response to NACT in terms of pCR in a large Danish breast cancer cohort, as well as the associations observed after stratification according to menopausal status and receptor status.

## Materials and methods

### Data sources

The Danish Breast Cancer Group (DBCG) [32] database registers all individuals with breast cancer in Denmark. From the DBCG database, we obtained data on patient, tumor, and treatment characteristics, as well as outcome data. Data were obtained on BMI from the Danish Anesthesia Database [33] and comorbidities from the National Patient Registry [34]. A unique identification number that is given to all Danish residents was used to merge all data at the individual level [35].

### Study population

**Fig. 1:**
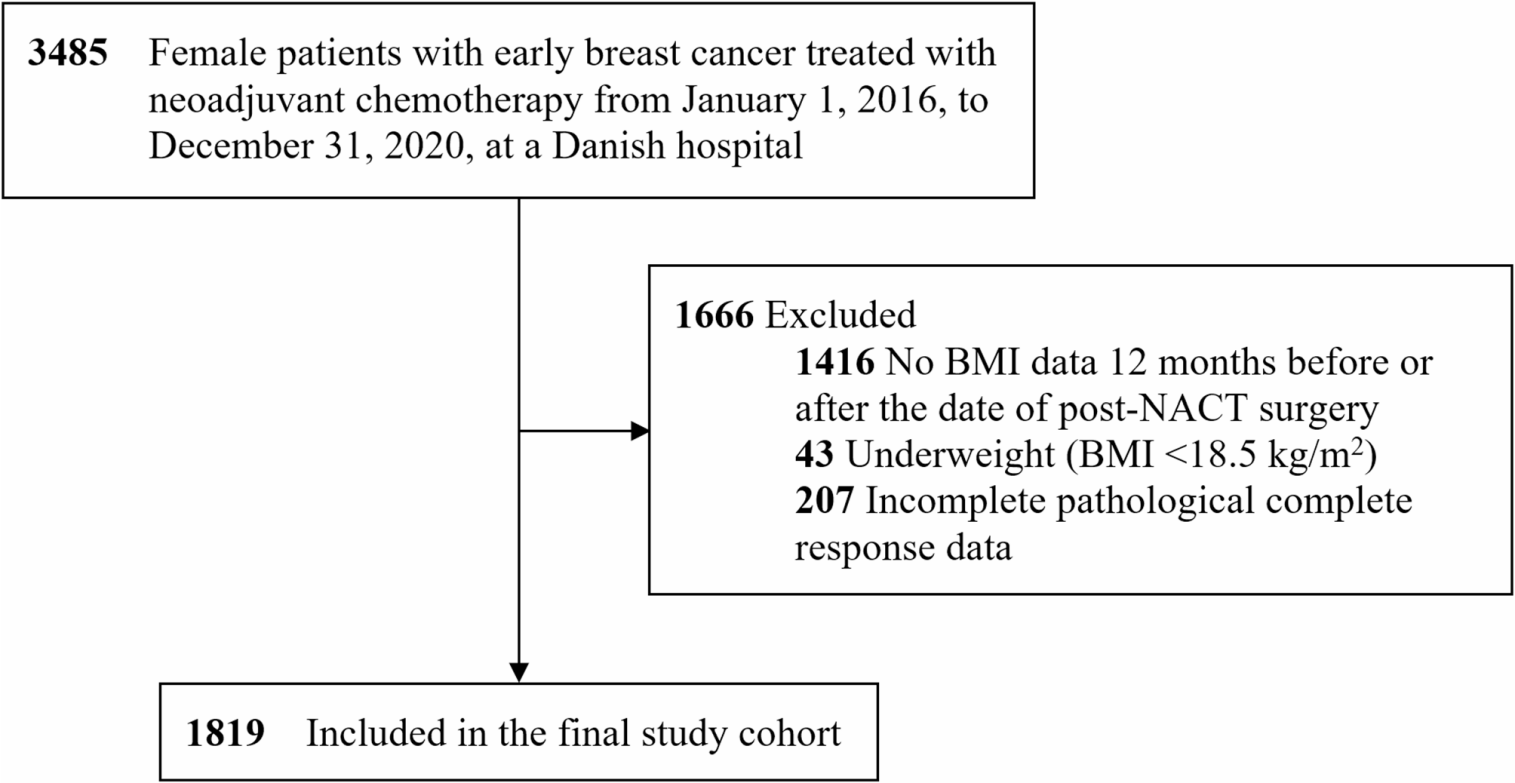
Flowchart of inclusion of patients in the final cohort. A total of 3485 patients with breast cancer treated with neoadjuvant chemotherapy and subsequent surgery in Denmark during the inclusion period were identified. We excluded 1416 patients who lacked BMI data from 12 months before or after the date of post-NACT surgery. Also, 43 patients with underweight were excluded. Of patients with BMI data 12 months before or after the date of surgery who did not have underweight, 207 patients had incomplete data on pathological complete response. In total, 1819 patients were included in the statistical analyses. Abbreviations: BMI Body Mass Index, NACT Neoadjuvant Chemotherapy.

This multi-center retrospective cohort study examined a population comprising women with operable or inoperable early breast cancer who were treated with NACT and subsequent surgery in Denmark between January 1, 2016, and December 31, 2020. We included all patients who met the inclusion criteria. A total of 3485 patients were identified in the DBCG database. We excluded patients who lacked BMI data from 12 months before or after the date of post-NACT surgery, patients with underweight (BMI < 18.5 kg/m^2^), and patients with incomplete data on tumor size and lymph node status from the post-NACT surgery (Fig. 1).

### Neoadjuvant treatment

Patients received NACT according to Danish guidelines in 2016 and 2020 [36]. Patients with stage T2N1 or lower were offered six series of EC-TAX pre-operatively (three series of epirubicin and cyclophosphamide, followed by three series of paclitaxel). Patients with a higher stage than T2N1 were offered eight series of EC-TAX pre-operatively. In HER2+ disease, trastuzumab and pertuzumab were added.

### Definitions of variables

#### Main exposure variable

BMI was assessed as a categorical variable (normal weight (BMI = 18.5-<25 kg/m^2^), overweight (BMI = 25-<30 kg/m^2^), and obesity (BMI ≥ 30 kg/m^2^)) and as a continuous variable (1-unit increase in BMI). We used the measurements of height and weight that were closest to the date of post-NACT surgery, including registrations from 12 months before or after the date of the surgery. Eight patients had duplicate measurements on the date closest to the date of surgery, and we used the mean of the two BMI registrations for these cases.

#### Covariates

*Patient characteristics.* Menopausal status (from the DBCG) and age corresponded to the time of breast cancer diagnosis (i.e., before NACT). The Charlson Comorbidity Index (CCI) [37] was used to assess comorbidities as a categorical variable (0, 1–2, and ≥ 3). Comorbidities registered up to 10 years before breast cancer diagnosis in the National Patient Registry were included.

*Tumor characteristics.* Tumor size before NACT was evaluated with ultrasound or magnetic resonance imaging and categorized according to the American Joint Committee on Cancer [38]. Lymph node status before NACT was evaluated as follows. If the radiologist located any suspicious lymph nodes during diagnostic assessment, fine needle aspiration (FNA) of the nodes was performed. Therefore, lymph node status was categorized as “clinical node-negative” if no suspicious lymph nodes were located or if the FNA showed no tumor cells and as “clinical node-positive” if the FNA showed the presence of tumor cells. Estrogen receptor (ER) status was assessed by immunohistochemistry, and tumors were classified as “ER-negative (ER-)” if no tumor cells expressed ER and as “ER-positive (ER+)” if 1% or more of the tumor cells expressed ER. HER2 expression was classified as positive (HER2+) or negative (HER2-) based on the criteria of the American Society of Clinical Oncology [39]. Both immunohistochemistry and the Fluorescence In Situ Hybridization (FISH)-ratio were used for the classification. The histological classification adhered to the World Health Organization’s 4th edition of Classification of Breast Tumors [40]. The Nottingham Group standardization was used for histological grade assessment [38].

*Treatment characteristics.* “Breast surgery type” refers to the type of post-NACT breast surgery performed (lumpectomy or mastectomy). “Mastectomy” refers to both patients who had mastectomy only and those who underwent mastectomy after lumpectomy. Axillary lymph node dissection refers to whether axillary lymph node dissection was performed post-NACT.

#### Outcomes

The achievement of pCR was dichotomized (yes/no), and the information was deduced from the pathological assessment of the breast tumor and lymph nodes from the post-NACT surgery. We defined pCR as total regression of invasive cancer in the breast and a lack of metastases in the regional lymph nodes (macrometastases, micrometastases, and clusters including isolated tumor cells). If any residual invasive tumor cells in the breast or lymph nodes were present, the patient was not considered to have achieved pCR.

### Statistical analysis

We conducted univariate and multivariable analyses using binary logistic regression models, which determined the odds ratios (ORs) of achieving pCR after NACT across BMI groups, using the normal weight group as reference, and per unit increase in BMI. In the multivariable analyses, we adjusted for confounders (age and menopausal status) based on a directed acyclic graph (Supplementary Fig. 1). Furthermore, we stratified analyses by ER, HER2, and menopausal status. We used scatter plots to visually test the assumption of linearity for BMI and age as continuous variables and their respective log odds, and the assumption of linearity was not violated.

## Results

The final study cohort consisted of 1819 patients with early breast cancer who were treated with NACT and subsequent surgery between 2016 and 2020 (Fig. 1). In total, 784 (43.1%) patients had normal weight, 585 (32.2%) had overweight, and 450 (24.7%) had obesity (Table 1). The majority of the patients had BMI measurement on the date of post-NACT surgery (90.4%). The median age was 52 years (interquartile range (IQR) 45–64 years), and 50 patients (2.7%) had a CCI of 3 or more.

**Table 1:** Patient characteristics of breast cancer patients treated with neoadjuvant chemotherapy included in the final cohort across body mass index groups

	Total (*N* = 1819)	Normal weight (BMI 18.5-<25 kg/m^2^) (*N* = 784)	Overweight (BMI 25-<30 kg/m^2^) (*N* = 585)	Obesity (BMI ≥ 30 kg/m^2^) (*N* = 450)
**Age (years)**,** median**	52 (45–64)	51 (44–66)	52 (46–65)	51 (45–62)
**Age (years)**,** categories**				
< 50	754 (41.5%)	340 (43.4%)	232 (39.7%)	182 (40.4%)
50–59	454 (25.0%)	167 (21.3%)	155 (26.5%)	132 (29.3%)
≥ 60	611 (33.6%)	277 (35.3%)	198 (33.8%)	136 (30.2%)
Missing	0	0	0	0
**Menopausal status**				
Premenopausal	889 (49.1%)	382 (48.9%)	287 (49.2%)	220 (49.1%)
Postmenopausal	923 (50.9%)	399 (51.1%)	296 (50.8%)	228 (50.9%)
Missing	7	3	2	2
**Charlson Comorbidity Index**				
0	1473 (81.0%)	657 (83.8%)	459 (78.5%)	357 (79.3%)
1–2	296 (16.3%)	105 (13.4%)	109 (18.6%)	82 (18.2%)
≥ 3	50 (2.7%)	22 (2.8%)	17 (2.9%)	11 (2.4%)
Missing	0	0	0	0

*Abbreviations: BMI Body Mass Index*

Patients with overweight or obesity had more comorbidities compared with patients with normal weight. Patients with overweight or obesity were more likely to have HER2- disease, and tumors of No Special Type (formerly known as ductal carcinoma) compared with patients with normal weight (Table 2). Patients with obesity had higher-grade tumors than patients with normal weight or overweight. Patients with overweight or obesity were more likely to undergo axillary lymph node dissection compared to patients with normal weight. Overall, the excluded patients (*N* = 1666) had similar characteristics to patients in the final study cohort (Supplementary Table 1).

**Table 2:** Tumor and treatment characteristics of breast cancer patients treated with neoadjuvant chemotherapy included in the final cohort across body mass index groups

	Total (*N* = 1819)	Normal weight (BMI 18.5-<25 kg/m^2^) (*N* = 784)	Overweight (BMI 25-<30 kg/m^2^) (*N* = 585)	Obesity (BMI ≥ 30 kg/m^2^) (*N* = 450)
**Tumor size pre-NACT**				
0–20 mm	396 (23.2%)	184 (25.0%)	131 (23.9%)	81 (19.3%)
21–50 mm	1096 (64.3%)	448 (60.9%)	358 (65.2%)	290 (69.0%)
> 50 mm	213 (12.5%)	104 (14.1%)	60 (10.9%)	49 (11.7%)
Missing	114	48	36	30
**Lymph node metastases pre-NACT**				
Clinical node-negative	1221 (67.2%)	520 (66.5%)	400 (68.4%)	301 (66.9%)
Clinical node-positive	596 (32.8%)	262 (33.5%)	185 (31.6%)	149 (33.1%)
Missing	2	2	0	0
**ER/HER2**				
ER+/HER2-	837 (47.6%)	326 (42.7%)	298 (52.7%)	213 (49.4%)
HER2+	548 (31.1%)	278 (36.4%)	146 (25.8%)	124 (28.8%)
ER-/HER2- (DNBC)	375 (21.3%)	159 (20.8%)	122 (21.6%)	94 (21.8%)
Missing	59	21	19	19
**Histological classification** ^**a**^				
No Special Type	1243 (84.4%)	515 (82.5%)	407 (85.5%)	321 (86.1%)
Lobular	84 (5.7%)	36 (5.8%)	29 (6.1%)	19 (5.1%)
Others/ unclassified	83 (5.6%)	34 (5.4%)	28 (5.9%)	21 (5.6%)
In situ	58 (3.9%)	37 (5.9%)	11 (2.3%)	10 (2.7%)
No tumor	5 (0.3%)	2 (0.3%)	1 (0.2%)	2 (0.5%)
Missing	346	160	109	77
**Histological grade**				
Not graded^b^	78 (4.5%)	32 (4.3%)	28 (5.1%)	18 (4.3%)
Grade 1	208 (12.1%)	95 (12.7%)	71 (12.9%)	42 (10.0%)
Grade 2	946 (55.0%)	408 (54.6%)	305 (55.4%)	233 (55.2%)
Grade 3	488 (28.4%)	212 (28.4%)	147 (26.7%)	129 (30.6%)
Missing	99	37	34	28
**Breast surgery type**				
Mastectomy	924 (50.8%)	399 (50.9%)	297 (50.8%)	228 (50.7%)
Lumpectomy	895 (49.2%)	385 (49.1%)	288 (49.2%)	222 (49.3%)
Missing	0	0	0	0
**Axillary lymph node dissection**				
No	896 (49.4%)	425 (54.5%)	273 (46.7%)	198 (44.0%)
Yes	918 (50.6%)	355 (45.5%)	311 (53.3%)	252 (56.0%)
Missing	5	4	1	0

*a: Tumors were classified through a combination of pathology reports on the tissue upfront and from the post-NACT surgery. Therefore*,* some tumors are classified as ”In situ” or ”No tumor*,*” as these were an option for the pathologists in the post-NACT surgery tumor tissue*

*b: A total of 78 patients’ tumors were not graded*,* for example*,* because No Special Type and non-lobular carcinomas were not graded during part of the cohort inclusion period or due to insufficient tumor tissue. “Not graded” was not considered a missing value in the multivariable models*

*Abbreviations: DNBC Double Negative Breast Cancer (ER-/HER2-)*,* ER Estrogen Receptor*,* FNA Fine-needle aspiration*,* HER2 Human Epidermal Growth Factor Receptor 2*,* NACT Neoadjuvant Chemotherapy*

In total, 22.9% (417/1819) of the patients had pCR (Table 3). Furthermore, pCR occurred for 25.8% of those with normal weight (202/784), 21.0% of those with overweight (123/585), and 20.4% of those with obesity (92/450). Compared with patients with normal weight, patients with overweight or obesity had 22% and 27% lower odds of pCR, respectively (overweight, OR_adj_=0.78 [95%CI = 0.60-1.00]; obesity, OR_adj_=0.73 [95%CI = 0.55–0.97]). For every 1-unit increase in BMI, the odds of pCR decreased by 2% (OR_adj_=0.98 [95%CI = 0.96-1.00]).

**Table 3:** Logistic regression presenting odds ratios of pathological complete response according to body mass index

	Number of pCRs/number of patients	Crude odds ratio (*N* = 1819)	Adjusted odds ratio based on a directed acyclic graph^a^ (*N* = 1812)
**Body Mass Index** ^**b**^			
Normal weight	202/784	Reference	Reference
Overweight	123/585	0.77 (0.59-0.99)	0.78 (0.60-1.00)
Obesity	92/450	0.74 (0.56–0.98)	0.73 (0.55–0.97)
Per unit increase		0.98 (0.96-1.00)	0.98 (0.96-1.00)
Total	417/1819		

^*a*^*Adjusted for age (continuous) and menopausal status.* ^*b*^*Normal weight = 18.5-<25 kg/m*^*2*^, *overweight = 25-<30 kg/m*^*2*^, *and obesity = ≥ 30 kg/m*^*2*^

*Abbreviations: BMI Body Mass Index*,* pCR pathological complete response*

Overall, 23.6% (210/889) of premenopausal patients and 22.3% (206/923) of postmenopausal patients achieved pCR. Postmenopausal patients with obesity were less likely to achieve pCR (OR_adj_=0.62 [95%CI = 0.41–0.94]) compared with postmenopausal patients with normal weight (Table 4). For every 1-unit increase in BMI, the odds of achieving pCR decreased by 3% for postmenopausal patients (OR_adj_=0.97 [95%CI = 0.94-1.00]). For premenopausal patients, a decrease in odds was found for patients with obesity (OR_adj_=0.86 [95%CI = 0.58–1.27]) compared with those with normal weight, but the association was less pronounced.

**Table 4:** Logistic regression presenting odds ratios of pathological complete response according to body mass index stratified by menopausal status

	Number of pCRs/number of patients	Crude odds ratio	Adjusted odds ratio based on a directed acyclic graph^a^
**Premenopausal**		*N* = 889	*N* = 889
Normal weight	99/382	Reference	Reference
Overweight	61/287	0.77 (0.54–1.11)	0.80 (0.55–1.15)
Obesity	50/220	0.84 (0.57–1.24)	0.86 (0.58–1.27)
Per unit increase		0.99 (0.96–1.02)	0.99 (0.96–1.02)
Total	210/889		
**Postmenopausal**		*N* = 923	*N* = 923
Normal weight	102/399	Reference	Reference
Overweight	62/296	0.77 (0.54–1.10)	0.76 (0.53–1.09)
Obesity	42/228	0.66 (0.44–0.98)	0.62 (0.41–0.94)
Per unit increase		0.97 (0.94-1.00)	0.97 (0.94-1.00)
Total	206/923		

^*a*^ *Adjusted for age (continuous)*

*Body Mass Index categories: Normal weight = 18.5-<25 kg/m*^*2*^, *overweight = 25-<30 kg/m*^*2*^, *and obesity = ≥ 30 kg/m*^*2*^

*Abbreviations: pCR pathological complete response*

In analyses stratified by receptor status, pCR occurred for 5.9% (49/837) of patients with ER+/HER2- tumors, 46.9% (257/548) of patients with HER2+ tumors, and 28.5% (107/375) of patients with ER-/HER2- tumors (i.e., Double Negative Breast Cancer (DNBC)) (Table 5). Patients with HER2+ tumors and obesity had 28% lower odds of achieving pCR compared to those with normal weight (OR_adj_=0.72 [95%CI = 0.47–1.12]). In ER+/HER2- we found no association (OR_adj_=0.97 [95%CI = 0.49–1.96]), and there was a less prominent association in DNBC tumors (OR_adj_=0.88 [95%CI = 0.49–1.57]), among patients with obesity compared to those with normal weight.

**Table 5:** Logistic regression presenting odds ratios of pathological complete response according to body mass index stratified by receptor status

	Number of pCRs/number of patients	Crude odds ratio	Adjusted odds ratio based on a directed acyclic graph^a^
**ER+/HER2-**		*N* = 837	*N* = 834
Normal weight	22/326	Reference	Reference
Overweight	13/298	0.63 (0.31–1.27)	0.64 (0.32–1.30)
Obesity	14/213	0.97 (0.49–1.94)	0.97 (0.49–1.96)
Per unit increase		0.99 (0.94–1.05)	0.99 (0.94–1.05)
Total	49/837		
**HER2+**		*N* = 548	*N* = 545
Normal weight	134/278	Reference	Reference
Overweight	72/146	1.05 (0.70–1.56)	1.09 (0.72–1.63)
Obesity	51/124	0.75 (0.49–1.15)	0.72 (0.47–1.12)
Per unit increase		0.98 (0.95–1.01)	0.98 (0.95–1.01)
Total	257/548		
**ER-/HER2- (DNBC)**		*N* = 375	*N* = 374
Normal weight	45/159	Reference	Reference
Overweight	38/122	1.15 (0.68–1.92)	1.17 (0.70–1.96)
Obesity	24/94	0.87 (0.49–1.55)	0.88 (0.49–1.57)
Per unit increase		0.99 (0.95–1.03)	0.99 (0.95–1.03)
Total	107/375		

^*a*^ *Adjusted for age (continuous) and menopausal status*

*Body Mass Index categories: Normal weight = 18.5-<25 kg/m*^*2*^, *overweight = 25-<30 kg/m*^*2*^, *and obesity = ≥ 30 kg/m*^*2*^

*Abbreviations: DNBC Double Negative Breast Cancer*,* ER Estrogen Receptor*,* HER2 Human Epidermal Growth Factor Receptor 2*,* pCR pathological complete response*

When assessing HER2+ tumors by ER status, patients with ER+/HER2+ tumors and obesity had lower odds of pCR compared with normal weight counterparts (OR_adj_=0.72 [95%CI = 0.42–1.23]) (Supplementary Table 2). For ER-/HER2+ disease, our data indicated lower odds of pCR for patients with obesity compared with patients with normal weight, but the estimate was imprecise (OR_adj_=0.79 [95%CI = 0.35–1.75]). For patients with overweight or obesity and ER+ tumors, regardless of HER2 status, we found a decrease in odds of pCR compared with patients with normal weight (overweight, OR_adj_=0.66 [95%CI = 0.46–0.94]; obesity, OR_adj_=0.70 [95%CI = 0.47–1.02]) (Supplementary Table 3).

## Discussion

In operable and inoperable early breast cancer patients treated with NACT, we found lower odds of pCR for patients with overweight or obesity compared with patients with normal weight. In postmenopausal patients, obesity was associated with lower odds of pCR, and to a lesser extent in premenopausal patients. Patients with HER2+ tumors and obesity had lower odds of pCR, while for ER+/HER2- and DNBC disease, no notable association was found.

Our results are in line with the majority of prior literature. Studies have reported lower odds of pCR among patients with overweight or obesity compared with patients with normal weight or underweight, or no association was observed [16, 19, 21–26, 30, 31, 41]. A meta-analysis by Wang et al. [16] (*N* = 18702) reported that patients with overweight or obesity were less likely to achieve pCR compared with patients with normal weight, which is consistent with our findings. Conversely, in the largest study so far (*N* = 4061), Fontanella et al. [30] reported that patients with overweight or obesity did not have lower odds of pCR compared with patients with normal weight in the multivariable analyses. Similarly, BMI was not associated with pCR status in a study from the USA by Kogawa et al. [41], which involved 4029 patients with breast cancer in stages I-III.

Analyses stratified by menopausal status show varying results [17, 22, 25, 27, 31]. Chen et al. [31] reported a lower pCR rate in Chinese postmenopausal women with overweight or obesity compared with normal weight or underweight counterparts (*N* = 307). Likewise, Guliyev et al. [27] found a lower pCR rate among postmenopausal patients with obesity compared with patients without obesity in a Turkish cohort (*N* = 191). Our results are consistent with those of Guliyev et al. [27] and Chen et al. [31] but differ from those of Farr et al. [17], who reported that postmenopausal patients with obesity had higher odds of pCR compared with patients without obesity (*N* = 120) in an Austrian cohort.

Results also differ in receptor stratified analyses [19, 25–27, 29–31]. In HER2+ disease, most previous studies have reported lower rates of pCR for patients with obesity [19, 25, 26, 29], but the association has varied with respect to HR status for the majority of the studies [19, 26, 29]. Warner et al. [29] (*N* = 1797) found higher odds of pCR for patients with obesity and ER-/HER2+ disease and lower odds for ER+/HER2+ disease. Wang et al. [26] (*N* = 978) found notably lower pCR rates for patients with obesity with HR-/HER2+ tumors, but not HR+/HER2+ tumors. Conversely, our results suggest that lower odds of pCR in HER2+ disease in patients with obesity do not depend on ER status. It should be noted that the results were imprecise, but our data indicated lower odds of pCR for patients with obesity in both ER+/HER2+ and ER-/HER2+ disease. Our results aligned with those of Chen et al. (*N* = 491) [25] who reported lower pCR rates for patients with obesity and HER2+ disease regardless of HR status. Regarding HR+/HER2- disease, Guliyev et al. [27] reported lower pCR rates for patients with obesity compared to patients with a BMI < 30 kg/m^2^. But similar to our findings, Wang et al. [26] and Warner et al. [29] did not observe lower pCR rates for patients with obesity compared to normal weight counterparts. As ER+ tumors respond poorly to NACT [13, 42], BMI could be a promising prognostic factor in these tumors, but our findings indicate that an association is only present in ER+/HER2+ disease. As a result, our data suggest that HER2+ appears to be a key factor in the association between lower pCR odds in cases of obesity compared to normal weight, while ER status has less significance.

Our study design differs from many previous studies, which could explain the difference in the results. Overall, the study populations were heterogeneous, as were BMI categories, and the reference estimates and exposures used were inconsistent between studies. Furthermore, different treatment regimens and treatment years could have influenced the outcomes as obesity modifies the pharmacokinetics of anticancer treatments, such as chemotherapy [43]. The results from Fontanella et al. [30] were based on two clinical trials (GeparQuattro [44] and GeparQuinto [45, 46]), which involved different treatment modalities compared to our study. In the study by Kogawa et al. [41], patients were treated between 1990 and 2013, so the results do not reflect current clinical practice. Like our study, Kogawa et al. [41] used a retrospective design. So far, the studies reporting menopause-stratified results have involved smaller cohorts from different countries [17, 22, 25, 27, 31], with the largest cohorts comprising 491 patients [22, 25], which may explain the widely ranging results. Also, the estimate references and exposures used varied from our study, as some included patients with overweight in the reference group [17, 27], and others grouped overweight and obesity as the exposure [22, 25, 31]. In studies reporting receptor-stratified analyses, Warner et al. [29] pooled patients from four prospective clinical trials, which contrasts with the design of our study. In the study by Wang et al. [26], chemotherapy dosage was not capped, which does not align with the NACT guidelines followed for our study cohort. Moreover, the anti-HER2 targeted therapy modalities highly varied across studies. In line with our study, the patients were treated with trastuzumab and pertuzumab in the studies by Wang et al. [26] and Chen et al. [25]. Conversely, Warner et al. [29] included patients treated with variations of the anti-HER2 treatment modalities, such as the addition of lapatinib, which was also the case for Di Cosimo et al. [19].

Different mechanisms may explain our observed lower odds of pCR in patients with obesity. As mentioned, obesity alters the pharmacokinetics of chemotherapy [43]. Furthermore, patients with obesity often experience reduced relative dose intensity (RDI), which is the ratio of chemotherapy delivered to the patient to the planned dose [47]. Patients receiving chemotherapy based on the actual body surface area experience more adverse side effects [48], so concerns over increased toxicity may result in capping of the dose [47]. In Denmark, dose capping can lead to reduced RDI in patients with obesity, potentially leading to undertreatment. In addition, many pathophysiological mechanisms may explain our results. First, adipokine signaling is altered in obesity [47, 49] and may affect the response to NACT according to Li et al. [50]. For instance, leptin and resistin have been suggested to induce chemoresistance through various pathways [50]. Second, obesity is associated with a chronic low-grade inflammatory state [47, 51], and pro-inflammatory cytokines may promote drug resistance [50]. Third, the extracellular matrix is remodeled in an obese state, causing tissue stiffness [22, 52], which may lead to a lower effect of chemotherapy [53]. Regarding the lower pCR odds in patients with obesity and HER2+ disease, Chen et al. [25] highlighted that patients with obesity may have a higher clearance of monoclonal antibodies like pertuzumab and trastuzumab. This could explain why we found lower pCR odds in patients with HER2+ disease and obesity, regardless of ER status.

Our findings contribute to the evidence on how BMI may affect NACT response in many ways. Ours is the largest study (when excluding meta-analyses) to report decreased odds of pCR in patients with obesity compared with patients with normal weight, as well as the largest study on BMI and NACT response in patients treated with newer treatment regimes. Furthermore, this is the largest study reporting ORs on BMI and pCR stratified by menopausal status and ER/HER2 status. In addition, a substantial number of studies have grouped overweight and obesity, but we separated the two BMI groups to further expand the knowledge on the matter.

As the response to NACT depends on the tumor subtype [13, 54], it is important to clarify how BMI may be used as a prognostic marker in patients with breast cancer treated with NACT. Our results suggest that obesity may be a prognostic marker for mainly HER2+ disease, as the association was less prominent in DNBC, and no association was found in ER+/HER2- disease. Future studies could focus on the role of BMI in NACT response across tumor subtypes and differentiate BMI categories in more detail to clarify how it may be used most appropriately and effectively as a prognostic marker. Furthermore, we suggest that future studies explore whether obesity-related conditions like altered adipokine levels may explain the association between obesity and lowered pCR odds compared to patients with normal weight.

### Limitations

Our study has limitations. First, the cohort is based on patients who underwent surgery after NACT, and we do not have information on the patients assigned to NACT who did not undergo surgery. Therefore, our study may be prone to selection bias. Moreover, we excluded 1666 patients, primarily due to missing BMI data. As a result, our results may not be representative of patients with breast cancer receiving NACT in general. Also, some of the subgroup analyses may have been underpowered to detect modest associations. Second, we did not have detailed information on actual NACT administered to the patients, so we relied on the treatment guidelines at the time in describing the NACT treatment. Third, BMI was recorded on the same date as when the patient underwent surgery, so no conclusions on causality can be made, and we can only confirm that an association was observed in our study. Fourth, BMI is a non-invasive and simple body measurement method, but it does not distinguish between adipose tissue and lean body mass, which could explain inconsistent results between studies [55]. Other anthropometric measurement methods may be more accurate in investigating the association between body composition and response to NACT [55].

## Conclusions

In conclusion, overweight and obesity were associated with lower odds of pCR compared with normal weight among patients with early breast cancer treated with NACT. The association could depend on the subtype, as the results varied across ER and HER2 status. The association seemed less affected by menopause status. Taken together, our findings are consistent with previous studies suggesting that BMI may be a prognostic marker of pCR.

## Electronic supplementary material

Below is the link to the electronic supplementary material.

Supplementary Material 1

## Data Availability

The data generated and/or analyzed during the current study are not publicly available due to individual privacy could be compromised for the study participants but are available from the corresponding author on reasonable request.

## Abbreviations

BMI: Body mass index
CCI: Charlson comorbidity index
DBCG: Danish breast cancer group
EC-TAX: Epirubicin, cyclophosphamide, and paclitaxel
ER: Estrogen Receptor
FISH: Fluorescence in situ hybridization
FNA: Fine-needle aspiration
HER2: Human epidermal growth factor receptor 2
HR: Hormone receptor
IQR: Interquartile range
NACT: Neoadjuvant chemotherapy
OR: Odds ratio
pCR: Pathological complete response
RDI: Relative dose intensity
