# Supplementary material for: The association between body mass index and neoadjuvant chemotherapy response in patients with breast cancer
Source: Breast Cancer Res. 2025 Jul 11;27:130. doi: 10.1186/s13058-025-02083-w (PMC12254981; doi:10.1186/s13058-025-02083-w)
Supplement: Supplementary file 1 — Supplementary Material 1 [file 13058_2025_2083_MOESM1_ESM.pdf]

Supplementary material

Supplementary Table 1: Characteristics of the excluded patients.

	**Total (N=1666)**
**Age (years), median**	52 (44-66)
**Age (years), categories**	
<50	697 (41.8%)
50-59	386 (23.2%)
≥60	583 (35.0%)
Missing	0
**Menopausal status**	
Premenopausal	818 (49.4%)
Postmenopausal	837 (50.6%)
Missing	11
**Charlson Comorbidity Index**	
0	1334 (80.1%)
1-2	282 (16.9%)
≥3	50 (3.0%)
Missing	0
**Tumor size pre-NACT**	
0-20 mm	305 (19.3%)
21-50 mm	1046 (66.0%)
>50 mm	233 (14.7%)
Missing	82
**Lymph node metastases pre-NACT**	
Clinical node-negative	1134 (68.1%)
Clinical node-positive	531 (31.9%)
Missing	1
**ER/HER2**	
ER+/HER2-	715 (44.8%)
HER2+	551 (34.5%)
ER-/HER2- (DNBC)	331 (20.7%)
Missing	69
**Histological classification**[a]	
No Special Type	1125 (82.6%)
Lobular	73 (5.4%)
Others/unclassified	118 (8.7%)
In situ	29 (2.1%)
No tumor	17 (1.2%)
Missing	304
**Histological grade**	
Not graded[b]	121 (8.1%)
Grade 1	139 (9.4%)
Grade 2	746 (50.2%)
Grade 3	480 (32.3%)
Missing	180
**Breast surgery type**	
Mastectomy	811 (48.7%)
Lumpectomy	855 (51.3%)
Missing	0
**Axillary lymph node dissection**	
No	892 (55.5%)

Yes	715 (44.5%)
Missing	59

a: Tumors were classified through a combination of pathology reports on the tissue upfront and from the post-NACT surgery. Therefore, some tumors are classified as "In situ" or "No tumor," as these were an option for the pathologists in the post-NACT surgery tumor tissue.
b: A total of 121 patients' tumors were not graded, for example, because No Special Type and non-lobular carcinomas were not graded during part of the cohort inclusion period or due to insufficient tumor tissue. "Not graded" was not considered a missing value in the multivariable models.
Abbreviations: BMI Body Mass Index, DNBC Double Negative Breast Cancer (ER-/HER2-), ER Estrogen Receptor, FNA Fine-needle aspiration, HER2 Human Epidermal Growth Factor Receptor 2, NACT Neoadjuvant Chemotherapy.

Supplementary Table 2: Logistic regression presenting odds ratios of pathological complete response according to body mass index in HER2+ disease by estrogen receptor status.

	Number of pCRs/number of patients	Crude odds ratio	Adjusted odds ratio based on a directed acyclic graph[a]
**ER+/HER2+**		N=380	N=378
Normal weight	81/188	Ref.	Ref.
Overweight	44/104	0.97 (0.60-1.57)	1.03 (0.63-1.68)
Obesity	32/88	0.75 (0.45-1.27)	0.72 (0.42-1.23)
Per unit increase		0.98 (0.95-1.02)	0.98 (0.94-1.02)
Total	157/380		
**ER-/HER2+**		N=165	N=164
Normal weight	52/88	Ref.	Ref.
Overweight	28/42	1.38 (0.64-2.99)	1.39 (0.64-3.02)
Obesity	19/35	0.82 (0.37-1.81)	0.79 (0.35-1.75)
Per unit increase		1.00 (0.94-1.06)	1.00 (0.94-1.06)
Total	99/165		

[a]Adjusted for age (continuous) and menopausal status.
Body Mass Index categories: Normal weight=18.5-<25 kg/m^2, overweight=25-<30 kg/m^2, and obesity=≥30 kg/m^2.
Abbreviations: ER Estrogen Receptor, HER2 Human Epidermal Growth Factor Receptor 2, pCR pathological complete response

Supplementary Table 3: Logistic regression presenting odds ratios of pathological complete response according to body mass index stratified by estrogen receptor status.

	Number of pCRs/number of patients	Crude odds ratio	Adjusted odds ratio based on a directed acyclic graph[a]
**ER-**		N=543	N=541
Normal weight	97/248	Ref.	Ref.
Overweight	66/164	1.05 (0.70-1.57)	1.07 (0.72-1.60)
Obesity	44/131	0.79 (0.51-1.23)	0.80 (0.51-1.24)
Per unit increase		0.99 (0.96-1.02)	0.99 (0.96-1.02)
Total	207/543		
**ER+**		N=1226	N=1221
Normal weight	103/515	Ref.	Ref.
Overweight	57/407	0.65 (0.46-0.93)	0.66 (0.46-0.94)
Obesity	46/304	0.71 (0.49-1.04)	0.70 (0.47-1.02)
Per unit increase		0.97 (0.95-1.00)	0.97 (0.95-1.00)
Total	206/1226		

[a]Adjusted for age (continuous) and menopausal status.
Body Mass Index categories: Normal weight=18.5-<25 kg/m^2, overweight=25-<30 kg/m^2, and obesity=≥30 kg/m^2.
Abbreviations: ER Estrogen Receptor, pCR pathological complete response

Supplementary Figure 1: Directed acyclic graph.

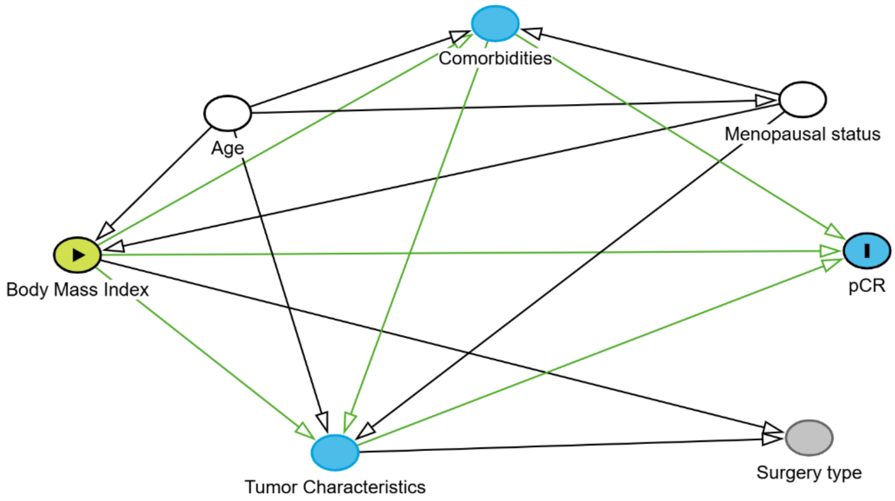

Supplementary Figure 1 legend: *Tumor size, lymph node metastases, receptor status (Estrogen receptor and Human Epidermal Growth Factor Receptor 2), grade, and classification are incorporated in "Tumor Characteristics." Comorbidities refer to comorbidities in the Charlson Comorbidity Index*(1). *"Surgery type" includes both the type of breast surgery and axillary surgery after neoadjuvant chemotherapy. Created on* *https://www.dagitty.net/dags.html. Abbreviations: pCR pathological complete response.*

References

1. Charlson ME, Pompei P, Ales KL, MacKenzie CR. A new method of classifying prognostic comorbidity in longitudinal studies: development and validation. J Chronic Dis. 1987;40(5):373–83.
